# High glucose-induced apoptosis in human coronary artery endothelial cells involves up-regulation of death receptors

**DOI:** 10.1186/1475-2840-10-73

**Published:** 2011-08-04

**Authors:** Shun-ichiro Kageyama, Hiroki Yokoo, Kengo Tomita, Natsuko Kageyama-Yahara, Ryo Uchimido, Naoyuki Matsuda, Seiji Yamamoto, Yuichi Hattori

**Affiliations:** 1 Department of Molecular and Medical Pharmacology, Graduate School of Medicine and Pharmaceutical Sciences, University of Toyama, Toyama 930-0194, Japan; 2 Division of Gastrointestinal Pathophysiology, Department of Bioscience, Institute of Natural Medicine, University of Toyama, Toyama 930-0194, Japan

## Abstract

**Background:**

High glucose can induce apoptosis in vascular endothelial cells, which may contribute to the development of vascular complications in diabetes. We evaluated the role of the death receptor pathway of apoptotic signaling in high glucose-induced apoptosis in human coronary artery endothelial cells (HCAECs).

**Methods:**

HCAECs were treated with media containing 5.6, 11.1, and 16.7 mM of glucose for 24 h in the presence or absence of tumor necrosis factor (TNF)-α. For detection of apoptosis, DNA fragmentation assay was used. HCAEC expression of death receptors were analyzed by the PCR and flow cytometry methods. Also, using immunohistochemical techniques, coronary expression of death receptors was assessed in streptozotocin-nicotinamide-induced type 2 diabetic mice.

**Results:**

Exposure of HCAECs to high glucose resulted in a significant increase in TNF-R1 and Fas expression, compared with normal glucose. High glucose increased TNF-α production by HCAECs and exogenous TNF-α up-regulated TNF-R1 and Fas expression in HCAECs. High glucose-induced up-regulation of TNF-R1 and Fas expression was undetectable in the presence of TNF-α. Treatment with TNF-R1 neutralizing peptides significantly inhibited high glucose-induced endothelial cell apoptosis. Type 2 diabetic mice displayed appreciable expression of TNF-R1 and Fas in coronary vessels.

**Conclusions:**

In association with increased TNF-α levels, the death receptors, TNF-R1 and Fas, are up-regulated in HCAECs under high glucose conditions, which could in turn play a role in high glucose-induced endothelial cell apoptosis.

## Background

Vascular complications are a leading cause of morbidity and mortality in diabetes. A wide variety of studies suggest that endothelial dysfunction and damage present pathophysiological early steps for the development of vascular complications [1,2]. A growing body of evidence indicates the close correlation between hyperglycemia and the abnormalities in endothelial function and morphology. High ambient glucose has been shown to disturb cell cycle [3,4], increase DNA damage [5], delay endothelial cell replication [3], and cause excessive cell death [3] in cultured human endothelial cells. More critically, that high glucose selectively triggers apoptosis in cultured endothelial cells has been demonstrated by subsequent studies from different laboratories [6-10]. An accelerated programmed cell death of retinal microvascular cells has been reported to occur *in situ *in human and experimental diabetic retinopathy [11,12]. Moreover, significant apoptosis of endothelial cells has been found in ventricular myocardial biopsies obtained from diabetic patients [13]. Several downstream pathways affected by hyperglycemia, such as oxidative stress [8-10,14,15] and glycation [16,17], are thought to stimulate apoptosis in endothelial cells. In contrast to its effect on endothelial cells, high glucose has been reported to inhibit apoptosis in coronary artery smooth muscle cells by up-regulating anti-apoptotic proteins [18]. Thus, the molecular events linking high glucose with the apoptotic machinery of endothelial cells may be far more complicated than being realized.

Apoptotic cell death in mammals can proceed by two distinct pathways that ultimately converge into a common pathway causing the activation of the effector enzymes caspases. The intrinsic pathway feeds cell death signals through the mitochondrion, which appears to act as a genetic damage sensor and monitor of metabolic status. Thus, injured mitochondria can release cytochrome *c *into the cytoplasm, where it can bind to apoptotic protease activating factor (APAF)-1 and activate caspase networks that induce apoptosis [19,20]. Recent report has shown that exposure to high glucose results in a decrease in the mitochondrial membrane potential and a release of cytochrome *c *in human umbilical vein endothelial cells [21]. Furthermore, high glucose treatment has been found to cause an increase in protein expression of Bax, whose complex formation is associated with cytosolic release of cytochrome *c*, in human aortic endothelial cells [22]. Proapoptotic signaling can also be mediated by the death receptor pathway. This extrinsic pathway transduces the signals of extracellular 'death ligands' which are members of the tumor necrosis factor (TNF) superfamily. After ligand binding to the intracellular death domain on the receptors, including TNF receptor 1 (TNF-R1) and Fas, the activated death receptors recruit adaptor proteins, which in turn recruit procaspase-8 into a proapoptotic complex termed the death-inducing signaling complex (DISC). Then, the initiator procaspase-8 is activated proteolytically into caspase-8 and further activates effector caspases along the common pathway of apoptosis [23,24]. One study has revealed that reduced T cell Fas ligand expression prevents diabetes in NOD mice [25], and several similar studies have focused on the importance of Fas in beta cell death and the development of diabetes [26,27], although evidence against a role of Fas has also been presented [28,29]. Nevertheless, data about a potential role of the death receptor apoptotic signaling pathway in the high glucose-induced apoptotic process in endothelial cells are still missing. Such information is important for understanding molecular mechanisms underlying high glucose-induced endothelial cell apoptosis.

In the present study, we initially examined whether high ambient glucose up-regulates expression of death receptors in cultured human coronary artery endothelial cells (HCAECs). We found that gene expression levels of the death receptors, TNF-R1 and Fas, were strikingly increased in endothelial cells under high-glucose conditions. Next, we investigated the possible involvement of the death receptor pathway of apoptotic signaling in high glucose-induced apoptosis in endothelial cells. Finally, we evaluated the possibility that coronary arteriolar endothelium may significantly express TNF-R1 and Fas in type 2 diabetic mice.

## Materials and methods

### Endothelial cell culture

HCAECs were purchased from Cambrex Bioscience (Walkersville, MD). Cells were used between passages 6 and 7 for experiments to avoid effects of *in vitro *cell aging. Cells were grown in endothelial growth media-2 (EGM-2; Cambrex) supplemented with growth factors and 5% (v/v) fetal bovine serum to 70-80% confluency. Cells were cultured in a humidified incubator at 37°C with a 5% CO_2 _atmosphere and the medium was changed every second day. To determine the effect of high glucose or TNF-α on cell apoptosis and death receptor expression, cells were incubated with both zero growth factors and zero hydrocortisone for 30 h, and they were then cultured for 24 h under different concentrations of glucose (5.6, 11.1 and 16.7 mM).

### Reverse transcription PCR (RT-PCR)

Total RNA was isolated from cells using standard procedure, including ISOGEN (Nippon Gene, Tokyo, Japan). The RNA was reverse-transcribed in a 20-μl reaction mixture that contained 5 U ReverScript^® ^III (Wako, Osaka, Japan) and 0.5 μg oligo(dT)12-18 primer (Invitrogen, Carlsbad, CA) at 42°C for 1 h, followed by 51°C for 30 min. The primer pairs for TNF-R1 (forward, 5'-GAG AGG CCA TAG CTG TCT GG-3'; reverse, 5'-GTT CCT TTG TGG CAC TTG GT-3'), for Fas (forward, 5'-TCA GTA CGG AGT TGG GGA AG-3'; reverse, 5'-CAG GCC TTC CAA GTT CTG AG-3'), for DR4 (forward, 5'-AGA GAG AAG TCC CTG CAC CA-3'; reverse, 5'-GTC ACT CCA GGG CGT ACA AT-3'), and for DR5 (forward, 5'-CAC CAG GTG TGA TTC AGG TG-3'; reverse, 5'-CCC CAC TGT GCT TTG TAC CT-3') yielded a 218-, 207-, 154-, and 221-bp PCR product, respectively. Human β-actin (forward, 5'-GGA CTT CGA GCA AGA GAT GG-3'; reverse, 5'-AGC ACT GTG TTG GCG TAC AG-3') was used as a reference gene. PCR was performed using an iCycler (Bio-Rad, Tokyo, Japan). The reaction mixture consisted of template cDNA, 0.2 μM of each primer, 300 μM dNTPs, 3 mM MgCl_2_, and 0.05 U/μl ExTaq DNA polymerase (Takara Shuzo, Ohtsu, Japan). PCR was performed for 32 cycles for each gene with denaturation at 95°C for 15 s, annealing at 61°C for 15 s, and extension at 72°C for 20 s. PCR products were quantified using NIH Image.

### Quantitative real-time PCR

Real-time PCR was performed in a final volume of 20 μl containing cDNA template and primers using Takara RNA PCR kit (Takara Shuzo) as described in the manufacture's manual. PCR amplification was carried out on the genomic DNA using the following primers: TNF-R1, forward 5'-AAC AGA ACA CCG TGT GCA CCT-3' and reverse 5'-AGT CCT CAG TGC CCT TAA CAT TCT C-3'; FAS, forward 5'-AGA GTA AAT GCA GTG GCA TGC TAA G-3' and reverse 5'-GGG TTA GCC TGT GGA TAG ACA TTT G-3'; DR4, forward 5'-GGA ACA CAG CAT GTC AGT GCA A-3' and reverse 5'-TGT CAC TCC AGG GCG TAC AAT C-3'; DR5, forward 5'-CAT CTA TGG ACA GGC TGG GAC A-3' and reverse 5'-CCC AAA CAG GGC TCA AGT TCA-3'; and actin, forward 5'-TGG CAC CCA GCA CAA TGA A-3' and reverse 5'-CTA AGT CAT AGT CCG CCT AGA AGC A-3'. The PCR program consisted of 95°C for 30 s for initial denaturation of DNA, followed by 40 cycles of 95°C for 5 s, 60°C for 34 s for annealing of primers, and 95°C for 1 min for elongation. The PCR product size for TNF-R1, FAS, DR4, DR5, and actin was 141, 150, 130, 96, and 186 bp, respectively; they were visualized after agarose gel electrodes and ethidium bromide staining. Actin served as an internal control for normalization.

### Immunocytochemistry and TUNEL staining

After being fixed with 4% paraformaldehyde for 15 min at room temperature, cells were kept in PBS at 4°C until staining. Unspecific binding sites were blocked in PBS containing 10% normal goat sera. The samples were treated with rabbit anti-human von Willebrand factor (vWF) antibody (Chemicon, Temecula, CA) at 1:100 dilution overnight at 4°C, carefully washed and stained with Alexa goat anti-rabbit secondary antibody (Invitrogen) at 1:500 dilution overnight at 4°C. For visualization of endothelial cells, cells were incubated with biotinylated *Griffonia simplicifolia *isolectin-B4 (Vector Laboratories, Burlingame, CA) overnight at 4°C, followed by an overnight incubation at 4°C with streptavidin (Alexa Fluor^® ^488 conjugate; Invitrogen). The nucleus was counterstained with Hoechst 33258 (Nakalai Tesque, Kyoto, Japan). Immunofluorescent images were observed under a Leica TCS-SP5 confocal sytem.

The terminal deoxynucleotide transferase-mediated dUTP nick end labeling (TUNEL) technique was conducted to detect apoptotic endothelial cells. The fluorescein *in situ *cell death detection kit (DeadEnd™ Fluorometric TUNEL System; Promega, Madison, WI) was used according to the manufacturer's instructions. The fluorosein-labeled cells undergoing apoptosis were recognized as a green fluorescent nucleus. The samples were analyzed using a fluorescence microscope.

### DNA fragmentation assay

To determine the extent of apoptosis, DNA fragmentation was assessed following the method by Arai et al. [30]. In brief, the cells were lysed in a lysis buffer (10 mM Tris, 1 mM EDTA, 0.2% Triton X-100, pH 7.5) and centrifuged at 13,000 × *g *for 10 min. Subsequently, each DNA sample in the supernatant and the pellet was precipitated in 12.5% trichloroacetic acid at 4°C and quantified using a diphenylamine reagent after hydrolysis in 5% trichloroacetic acid at 90°C for 20 min. After the absorbance of the supernatant and the pellet was determined spectrophotometrically at a wavelength of 600 nm, the percentage of fragmented DNA in each sample was calculated as the amount of DNA in the supernatant divided by the total DNA for that sample (supernatant plus pellet). The average percentage of fragmented DNA in control samples was 2.7%.

### Flow cytometry analysis

Cells were washed with FACS buffer (PBS, 1% bovine serum albumin, 0.2% NaN_3_), stained with FITC-labeled anti-human TNF-R1 antibody (clone MABTNFR1-B1; BD Pharmingen, San Diego, CA) for 30 min at 4°C, followed by phycoerythrin-labeled goat polyclonal anti-mouse IgG (clone ab74490, Abcam, Cambridge, England). Propidium Iodide (Sigma-Aldrich, St. Louis, MO) was used to discriminate between dead and live cells. Flow cytometric analysis was performed using FACSCalibur and Cell QuestPro software version 6.0 (BD Biosciences, Franklin Lakes, NJ). TNF-R1 expression was determined using mean fluorescence intensity (MFI), with vehicle-treated cells as the baseline for all comparisons.

### Enzyme immunoassay for TNF-α

TNF-α concentrations were determined in cell culture supernatants by enzyme-linked immunosorbent assay (ELISA) (BioVender, Modřice, Česká republika). Sensitivity of the assay was 4 pg/ml for TNF-α. No cross reactivity was observed with other human inflammatory cytokines including various interleukins. Optical density was measured at 450 nm by use of a microplate reader. Plasma levels of TNF-α were measured by the use of a commercially available enzyme-linked assay kit (Shibayagi Co. Ltd., Shibukawa, Japan) according to the manufacture's instructions.

### Mouse model of type 2 diabetes

The animal study was carried out as approved by the Animal Care and Use Committee of University of Toyama. Male ICR mice, 5-weeks old, received an intraperitoneal injection of 1.5 g/kg body weight of nicotinamide dissolved in saline 15 min before a tail-vein injection of streptozotocin (200 mg/kg; Sigma-Aldrich) dissolved in a citrate buffer solution (0.1 M citric acid and 0.2 M sodium phosphate, pH 4.5), following the protocol previously reported [31,32]. Control mice received the vehicles of both substances. The experimental model prepared here was devised more than a decade ago [33]. Its diabetic syndrome appears to share a number of features with human type 2 diabetes [31-33]. Animals were diabetic for 9 weeks before heart harvest. Only animals with glycemia levels above 300 mg/dl were used for experiments.

### Immunofluorescence and confocal analysis

Mouse hearts were harvested, fixed with 4% buffered formalin solution, immersed in sucrose solutions, dipped into OCT compound (Sakura Finetechnical, Tokyo, Japan), and frozen at -20°C. The embedded tissues were then sectioned at a thickness of 30 μm and air dried. For immunohistochemical detection of the target molecules, the tissue sections were exposed to the fluorescent secondary antibody after incubation with the suitable primary antibody according to the method in our previous study with minor modification [34]. Thus, rehydrated sections were incubated with the primary antibody, anti-mouse CD31 (BD Pharmingen, Franklin Lakes, NJ), anti-mouse TNF-R1 (BD Pharmingen), or anti-mouse Fas (Merck KGaA, Darmstadt, Germany), overnight at 4°C. The antibodies were used at 1:100 dilution, followed by extensive washes with PBS and incubation with the secondary antibodies conjugated to high quality fluophores, including Alexa Fluor 568, Alexa Fluor 488, and DyLight 488, at 1:500 dilution overnight at 4°C. The nucleus was counterstained with Hoechst 33258 (Nacalai Tesque, Kyoto, Japan). Immunofluorescent images were observed under Leica TCS-SP-5 confocal system (Leica, Wetzlar, Germany).

### Statistical analysis

All experiments were performed at least three times. Data are presented as means ± SE. Statistical assessment of the data was made by one- or two-way ANOVA, and then differences among groups were analyzed by Turkey's multiple comparison test, with level of significance set at *P *< 0.05.

## Results

### High glucose-induced endothelial cell apoptosis

**Figure 1:**
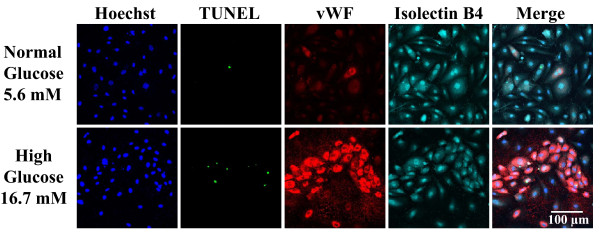
**Effects of high glucose on expression of the inflammatory marker vWF and TUNEL staining-apoptosis in HCAECs**. Cells were treated with 16.7 mM glucose for 24 h. Endothelial cell apoptosis was labeled by the TUNEL reaction (*green*). vWF (*red*) and isolectin B4 (*turquoise*) were visualized by fluorescence microscopy. Isolectin B4 was used as a marker for endothelial cells. Nuclei were counterstained with Hoechst (*blue*).

The blood clotting protein vWF is synthesized and secreted by endothelial cells. It is a useful maker for endothelial cells, but appears to represent the presence of vascular, especially endothelial, damage and play an important role in thrombosis and inflammation [35]. Immunofluorescent staining for vWF showed that its expression was markedly increased in HCAECs under high glucose conditions (Figure 1). However, it should be noted that this was observed in HCAECs at passage 6~7 (used in this study) but not at passage 2, although there was no obvious difference in morphology under microscope observation up to passage 10 (data not shown).

**Figure 2:**
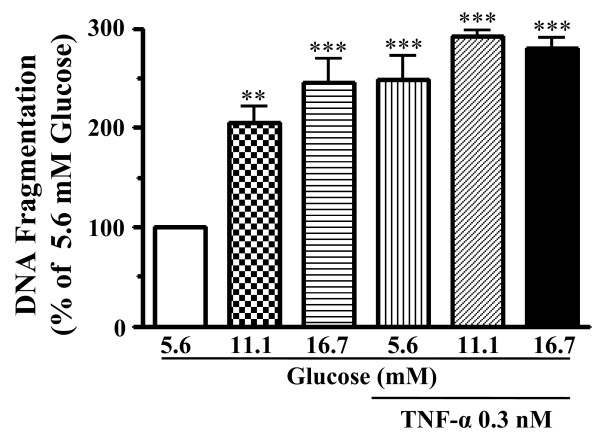
**Abilities of high glucose (11.1 and 16.7 mM) and TNF-α (0.3 nM) to induce apoptosis in HCAECs**. The DNA fragmentation was assayed as a biochemical hallmark of apoptosis. Values are expressed as mean ± SE (*n *= 3). **P < 0.01, ***P < 0.001 versus normal glucose (5.6 mM) without TNF-α.

When cells were labeled with an *in situ *TUNEL assay to detect apoptotic cells, exposure to high glucose resulted in a striking appearance of TUNEL-positive cells (Figure 1). The merge image showed that a positive TUNEL reaction was observed only in vWF positive cells. To quantify high glucose-induced apoptosis, we measured DNA fragmentation in HCAECs. As shown in Figure 2, exposure to high glucose increased DNA fragmentation in a manner dependent on the concentration of glucose. Treatment with TNF-α increased DNA fragmentation to the same extent as 16.7 mM glucose did. Combining TNF-α and high glucose exhibited no additive effect compared with high glucose alone.

To rule out an osmotic effect, we added 11 mM mannitol to 5.6 mM glucose. It was confirmed that mannitol was without effect on either vWF expression or cell apoptosis induction (data not shown).

### Death receptor expression under high glucose

**Figure 3:**
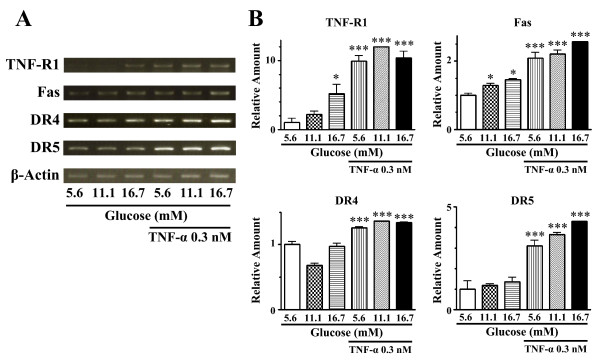
**Abilities of high glucose (11.1 and 16.7 mM) and TNF-α (0.3 nM) to up-regulate gene expression levels of death receptors in HCAECs**. The mRNA levels of TNF-R1, Fas, DR4, and DR5 were analyzed by RT-PCR (A) and real-time PCR (B). Actin was used as a reference gene for normalization of quantitative real-time PCR. Values are expressed as mean ± SE (*n *= 3). *P < 0.05, **P < 0.01, ***P < 0.001 versus normal glucose (5.6 mM) without TNF-α.

To determine how high glucose environment regulates gene expression of death receptors in human endothelial cells, the mRNA expression levels of TNF-R1, Fas, DR4, and DR5 in HCAECs cultured for 24 h under high glucose conditions were evaluated by PCR analysis (Figure 3). High glucose exposure resulted in a concentration-dependent increase in mRNA expression of TNF-R1. Mannitol, used as an osmolarity control, had no effect on TNF-R1 mRNA expression (data not shown). Another death receptor, Fas, was significantly increased under high glucose conditions, although the extent of up-regulation was less than that seen for TNF-R1. In contrast, mRNA expression levels of DR4 and DR5, both of which mediate TRAIL (TNF related apoptosis inducing ligand)-induced cell death, under high glucose did not significantly differ from those under normal glucose conditions.

Treatment with TNF-α (0.3 nM) strikingly up-regulated mRNA expression levels of all death receptors (Figure 3). High glucose-induced up-regulation of TNF-R1 and Fas mRNA expression was concealed in the presence of TNF-α.

**Figure 4:**
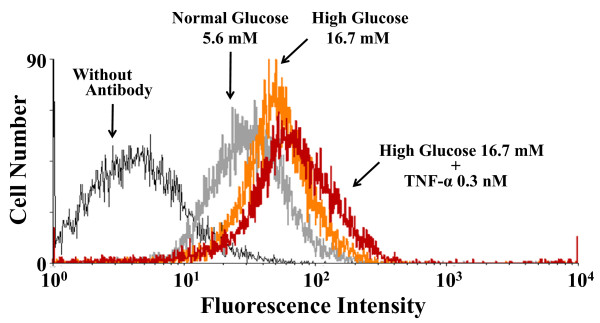
**Representative histograms of HCAECs expressing TNF-R1**. Surface expression of TNF-R1 was analyzed by flow cytometry using FITC-labeled anti-human TNF-R1 antibody. Cells were treated with 16.7 mM glucose in the presence or absence of 0.3 nM TNF-α for 24 h. Similar results were obtained with two additional experiments.

We characterized surface expression of TNF-R1 on HCAECs using fluorescence flow cytometry (Figure 4). Histograms generated by flow cytometry software showed that a reproducible shift in TNF-R1 surface expression compared to the mannitol-added control was observed with exposure to high glucose. When TNF-α was further treated under high glucose conditions, the combined effect on surface expression of TNF-R1 was not so pronounced.

### Effect of TNF-R1 neutralizing peptides on high glucose-induced apoptosis

**Figure 5:**
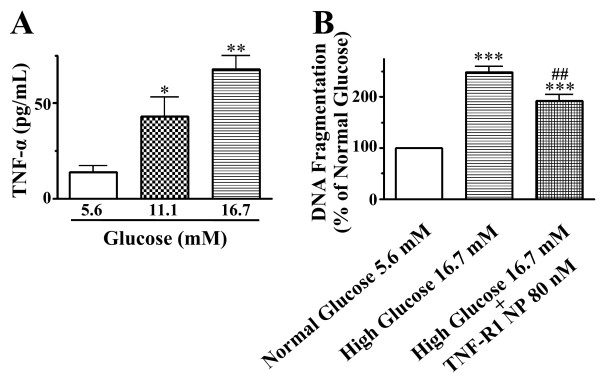
**TNF-α production and TNF-R1 neutralizing peptides-sensitive apoptosis in HCAECs under high glucose conditions**. A: Effect of high glucose on TNF-α levels in cell culture supernatants, analyzed by ELISA. Values are expressed as mean ± SE (*n *= 3). **P < 0.05, ***P < 0.01 versus normal glucose (5.6 mM). B: Effect of TNF-R1 neutralizing peptides on high glucose-induced endothelial cell apoptosis. TNF-R1 neutralizing peptides (80 nM) were given in HCAECs treated with 16.7 mM glucose for 24 h. The DNA fragmentation was assayed as a biochemical hallmark of apoptosis. Values are expressed as mean ± SE (*n *= 3). ***P < 0.001 versus control (normal glucose); ##*P *< 0.01 versus high glucose treatment alone.

TNF-α concentrations in cell culture supernatants were measured with ELISA. ELISA analysis showed that, under two high glucose conditions (11.1 and 16.7 mM), TNF-α levels were increased about 3.5- and 5.5-fold, respectively, compared to normal glucose medium (Figure 5A).

When TNF-R1 neutralizing peptides (80 nM) were given during exposure to high glucose, DNA fragmentation caused by high glucose was significantly inhibited by 23% (Figure 5B). We confirmed that the presence of TNF-R1 neutralizing peptides (20, 40, and 80 nM) inhibited TNF-α (0.3 nM)-induced DNA fragmentation by 15, 38, and 52%, respectively.

### Coronary expression of TNF-R1 and Fas in type 2 diabetic mice

**Figure 6:**
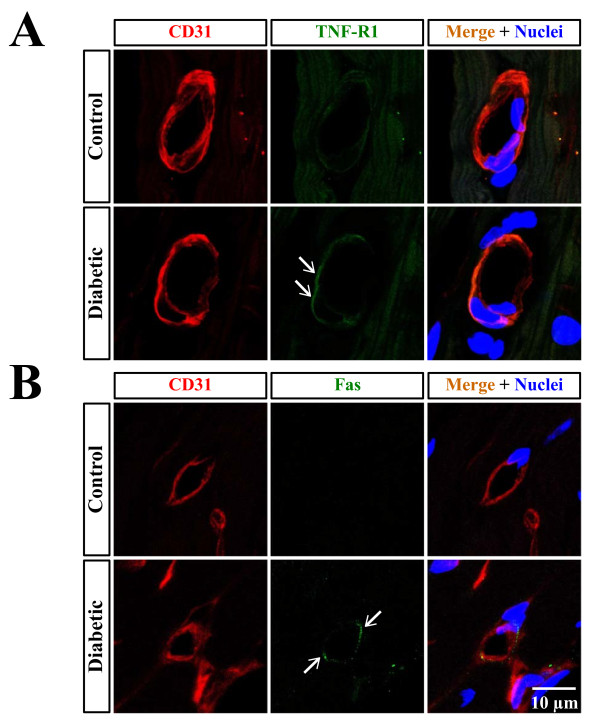
**Immunofluorescent images for TNF-R1 (A) and Fas (B) (*green*, white arrows) in the left ventricular cross-sections from control and nicotinamide-streptozotocin-induced diabetic mice**. Coronary arterial endothelial cells are stained with anti-CD31 antibody (*red*). Overlap images indicate that both TNF-R1 and Fas are present on coronary arterial endothelial cells. Nuclei were counterstained with Hoechst (*blue*). Shown are representative micrographs from two independent experiments in which the same results were obtained.

We generated type 2 diabetic mice which were administered streptozotocin and partially protected with nicotinamide. The non-fasting plasma glucose levels were 149 ± 28 mg/dl (n = 4) and 583 ± 90 mg/dl (n = 5) for control and diabetic mice, respectively (*p *< 0.05). Plasma TNF-α levels were much higher in diabetic than in control mice (27.2 ± 5.4 vs 6.5 ± 2.7 pg/ml, *p *< 0.05). Immunofluorescence staining for TNF-R1 and Fas in the left ventricular cross-sections is shown in Figure 6. Control mice had no positive staining for these death receptors. On the other hand, appreciable positive staining was found in diabetic mice. Overlap images indicated that both TNF-R1 and Fas were expressed on coronary arterial endothelium.

## Discussion

A growing body of evidence has shown that hyperglycemia triggers various vascular endothelial dysfunctions during the development of diabetes. Among them, high glucose-induced endothelial cell apoptosis has been noted in the pathogenesis of the acceleration of atherosclerosis associated with diabetes [6,9]. In the present study, we also demonstrated that, in exposure to high glucose for 24 h, HCAECs can undergo apoptotic cell death. Analysis of *in situ *TUNEL assay and DNA fragmentation provides clear experimental evidence for high glucose-induced apoptosis in HCAECs.

Previous works have indicated that high glucose treatment increases Bax protein, an accelerator of apoptosis, which in turn releases cytochrome *c *from mitochondria. The released cytochrome *c *into cytoplasm can bind to Apaf-1, resulting in activation of the caspase cascade that induces apoptosis [21,22]. Thus, cytochrome *c *released from mitochondria has been thought to be important in high glucose-induced endothelial cell apoptosis. We found that gene and surface protein expression levels of TNF-R1 and Fas were highly up-regulated in HCAECs under high glucose conditions. High glucose-triggered death receptor up-regulation in human endothelial cells was not due to changes in osmolarity, because no increase in these death receptors was seen in HCAECs treated with the osmotic control mannitol. Furthermore, high ambient glucose did not lead to a significant up-regulation of DR4 and DR5 in HCAECs, both of which are known to be receptors for TRAIL [36]. Our present findings would suggest the pathophysiological significance of the ligand-specific death receptor apoptotic signaling pathway, in addition to the intrinsic mitochondrial-mediated apoptotic signaling pathway, in high glucose-induced human endothelial cell apoptosis.

Apparent increases in plasma concentrations of proinflammatory cytokines, including TNF-α, seem to be seen in subjects with obesity and insulin resistance [37]. It has been shown that expression of TNF-α as well as other proinflammatory cytokines and chemokines is regulated by glucose in human monocytes and monocytic cell lines [38-40]. Thus, high glucose treatment activates these cells and induces an increase in gene expression of TNF-α. We found that high glucose exposure of HCAECs resulted in a marked increase in TNF-α levels in cell culture supernatants. High glucose-induced high expression of TNF-α has also been demonstrated in cultured cardiomyocytes of neonatal rats [41]. To the best of our knowledge, however, this is the first to demonstrate the high glucose-induced increases in TNF-α production by human endothelial cells. In light of the up-regulation of TNF-R1, a membrane receptor that mediates cytotoxicity elicited by TNF-α, in HCAECs under high glucose conditions, we interpret this finding to indicate that the TNF-α system may play a key part in high glucose-induced endothelial cell apoptosis.

Treatment with TNF-α in normal glucose medium caused a marked up-regulation of mRNA levels of death receptors in HCAECs. Therefore, TNF-α appears to be a major determinant of high glucose-induced up-regulation of TNF-R1 and Fas expression in endothelial cells. Consistent with this is the finding that high glucose resulted in no further up-regulation of TNF-R1 and Fas mRNA expression in the presence of TNF-α. However, high glucose-induced up-regulation of TNF-R1 and Fas cannot be solely attributed to the increased TNF-α production by endothelial cells exposed to high glucose. Endothelial expression of DR4 and DR5 mRNAs was strikingly up-regulated by TNF-α treatment but not by high glucose exposure. Experimental evidence for the regulatory mechanism(s) by which TNF-α can increase gene expression of these TRAIL receptors awaits further study.

A potential role of the TNF-α system in high glucose-induced human endothelial cell apoptosis was tested with TNF-R1 neutralizing peptides when given at a concentration of 80 nM. TNF-R1 neutralizing peptides showed a significant inhibition of high glucose-induced DNA fragmentation in HCAECs. This suggests that the TNF-α system is involved in human endothelial cell apoptosis induction under high glucose conditions. However, the inhibitory effect of TNF-R1 neutralizing peptides was less than 40%. It should be noted that the dose of TNF-R1 neutralizing peptides employed in this study caused >50% inhibition of 0.3 nM TNF-α-induced DNA fragmentation in HCAECs. Alternatively, the Fas/Fas ligand system as well as the intrinsic mitochondrial-mediated apoptotic signaling pathway may be additional mechanisms for the remaining effect of high ambient glucose to induce human endothelial cell apoptosis. The involvement of the Fas/Fas ligand system in high glucose-induced apoptosis of human islets has been demonstrated using the antagonistic anti-Fas antibody ZB4 [42]. It may be considered that dual treatment with TNF-R1 neutralizing peptide and ZB4 could suppress high glucose-induced DNA fragmentation more efficiently than TNF-R1 neutralizing peptide alone.

Exposure of endothelial cells to excess glucose has been reported to induce inflammation [43,44]. The underlying mechanisms are thought to be related to accumulation of proinflammatory intermediates or by-products such as reactive oxygen species. In this study, the inflammatory marker vWF was strongly expressed in HACECs when treated with high glucose. Importantly, the merge image showed that a positive TUNEL reaction was observed only in vWF positive cells. This implies that apoptosis may be specifically induced in inflammatory endothelial cells. In endothelial cells under high glucose conditions, increased inflammation could lead to enhanced apoptosis. Although the inflammatory process involves the activation of the transcription factor nuclear factor-κB (NF-κB) that is often associated with a predominantly antiapoptotic role through its ability to up-regulate cytoprotective gene products, the induction of the potential regulator that globally induces expression of proapoptotic genes may represent a mechanism to overcome NF-κB-associated antiapoptosis [45].

Finally, our immunofluorescence study found that chronic type 2 diabetic mice showed appreciable expression of TNF-R1 and Fas in the intimal surface of coronary arterioles. No positive staining for these death receptors was obtained in age-matched control mice. Circulating TNF-α levels were elevated in diabetic mice as reported in diabetic patients as well as in streptozotocin-induced diabetic rats [46,47]. Also, Fas ligand, whose expression is limited to cells of the immunosystem, has been shown to be significantly up-regulated in diabetic rat neutrophils [48]. Therefore, induction of death receptor expression in coronary arteriolar endothelium may promote coronary endothelial cell apoptosis during chronic diabetes. It is striking that there is a clinical study showing increases in apoptosis of cardiomyocytes, endothelial cells, and fibroblasts in specimens of myocardial tissues from diabetic patients [11].

In conclusion, the present study showed that the death receptors, TNF-R1 and Fas, are up-regulated in HCAECs under high glucose conditions. This up-regulation of death receptor expression, coupled with increased TNF-α secretion, could promote endothelial cell apoptosis, which is likely to contribute to coronary arterial endothelial dysfunction and the development of ischemic heart disease in diabetes.

## Conflicts of interests

The authors declare that they have no competing interests.

## Authors' contributions

SK, HY, NM, and YH designed research; SK, HY, KT, NK-Y, SY, and RY performed research; SK, HY, and KT analyzed data; SK, HY, and YH wrote the paper. All authors have read and approved submission of the final manuscript.
